# Impact of the Zinc Antiviral Protein on the Genomic Composition of RNA Viruses Infecting Vertebrates

**DOI:** 10.1093/molbev/msaf135

**Published:** 2025-06-04

**Authors:** Diego Simón, Daniela Megrian, Hunter K Walt, Pilar Moreno, Héctor Musto, Federico G Hoffmann, Gonzalo Moratorio

**Affiliations:** Laboratorio de Virología Molecular, Facultad de Ciencias, Universidad de la República, Montevideo, Uruguay; Laboratorio de Evolución Experimental de Virus, Institut Pasteur de Montevideo, Montevideo, Uruguay; Laboratorio de Genómica Evolutiva, Facultad de Ciencias, Universidad de la República, Montevideo, Uruguay; Unidad de Bioinformática, Institut Pasteur de Montevideo, Montevideo, Uruguay; Department of Biochemistry, Nutrition and Health Promotion, Mississippi State University, Mississippi State, MS 39762, USA; Laboratorio de Virología Molecular, Facultad de Ciencias, Universidad de la República, Montevideo, Uruguay; Laboratorio de Evolución Experimental de Virus, Institut Pasteur de Montevideo, Montevideo, Uruguay; Centro de Innovación en Vigilancia Epidemiológica, Institut Pasteur de Montevideo, Montevideo, Uruguay; Laboratorio de Genómica Evolutiva, Facultad de Ciencias, Universidad de la República, Montevideo, Uruguay; Department of Biochemistry, Nutrition and Health Promotion, Mississippi State University, Mississippi State, MS 39762, USA; Institute for Genomics, Biocomputing and Biotechnology, Mississippi State University, Mississippi State, MS 39762, USA; Laboratorio de Virología Molecular, Facultad de Ciencias, Universidad de la República, Montevideo, Uruguay; Laboratorio de Evolución Experimental de Virus, Institut Pasteur de Montevideo, Montevideo, Uruguay; Centro de Innovación en Vigilancia Epidemiológica, Institut Pasteur de Montevideo, Montevideo, Uruguay

**Keywords:** CpG bias, dinucleotide frequencies, genome compositional properties, RNA viruses, zinc-finger antiviral protein

## Abstract

The composition of viral genomes, influenced by host-specific biases, offers insights into their evolutionary history. Vertebrate cells counter viral infection with interferons (IFNs) that activate IFN-stimulated genes, including the zinc-finger antiviral protein (ZAP), which binds CpG-rich single-stranded viral RNA (ssRNA). We trace the origin of ZAP along the vertebrate phylogeny and highlight its earlier emergence than previously described. Our analysis of ZAP orthologs shows that ZAP originated from a PARP12-like ancestor in the last common ancestor of tetrapods and lungfishes, more than 400 million years ago. Amphibian ZAP shares structural domains with its mammalian counterpart, though it typically lacks the C-terminal CAAX-box motif. The conserved RNA-binding domain in lungfish and tetrapod suggests an early functional reassignment. Subsequently, we found that CpG suppression in ssRNA viral genomes increases with the phylogenetic proximity of hosts to mammals, with amniote-infecting viruses showing the strongest bias, likely reflecting adaptation to ZAP-mediated immunity. These findings suggest that ZAP’s evolutionary steps include gene duplication in jawed vertebrates, structural adaptations in sarcopterygians, and membrane targeting capabilities in an early tetrapod, reflecting the complex coevolution of host antiviral defenses and viral evasion strategies.

## Introduction

Viral genome composition, shaped by host compositional biases, provides insights into viral origin and evolution (Kapoor et al. 2010). In vertebrates, viral infections elicit the production of interferons (IFNs), which activate a cascade of immune responses mediated by IFN-stimulated genes (ISGs) (Isaacs and Lindenmann 1957; Schultz et al. 2004; Schoggins and Rice 2011). Among these, the zinc-finger antiviral protein (ZAP) is a key ISG that restricts the replication of several viruses, initially characterized for its role against retroviruses (Gao et al. 2002). ZAP belongs to the Poly ADP-Ribose Polymerase (PARP) superfamily, known for its involvement in DNA repair, transcriptional regulation, cell proliferation, and immune responses (Amé et al. 2004). Specifically, ZAP, also referred to as zinc-finger CCCH-type antiviral protein 1 (ZC3HAV1), contains CCCH-type zinc-finger domains that bind CpG-rich single-stranded viral RNA (ssRNA), effectively inhibiting viral replication (Takata et al. 2017; Meagher et al. 2019).

The importance of CpG dinucleotides extends beyond viral RNA. CpG was first noted as an underrepresented dinucleotide in calf thymus DNA (Josse et al. 1961), a pattern later observed in various tissues and attributed to DNA methylation (Swartz et al. 1962; Bird 1980). The hypothesis that ssRNA viruses mimic host genomic features, including CpG suppression, was first proposed by Greenbaum et al. (2008). This concept, known as host-driven CpG suppression, remains pivotal in understanding viral evolution and host-virus interactions, as cells display antiviral mechanisms to recognize nonself ssRNA enriched in CpG dinucleotides (Schlee and Hartmann 2016; Kustin and Stern 2021). In particular, human ZAP is known to inhibit a broad range of viruses, including representatives of different classes (de Andrade and Cirne-Santos 2023); e.g. positive-sense, ssRNA(+) (Bick et al. 2003); negative-sense, ssRNA(−) (Müller et al. 2007), and retro-transcribing (RT), RT-ssRNA(+) (Gao et al. 2002). Recently, ZAP has been proposed to be a gene specific to tetrapods (Gonçalves-Carneiro et al. 2021).

Here, we combined homology searches with phylogenetic, structural, and synteny analyses to trace the origin and evolutionary history of ZAP. We then estimated its impact on the genomes of their cognate ssRNA viruses, as ZAP is a broadly present restriction factor in different hosts. To do so, we analyzed orthologs of ZAP and close paralogs in all major vertebrate lineages, and we explored the CpG content of their ssRNA infecting viruses. Our findings indicate that ZAP originated in the last common ancestor of lungfishes and tetrapods through the duplication event of a PARP12-like gene and that its antiviral properties evolved a new function in a stepwise manner. This process started with the acquisition of a ZAP-like RNA-binding domain (RBD; ZnF_C3H1) in the common ancestor of lungfishes and tetrapods around 408 million years ago, followed by the acquisition of the helix-turn-helix domain (HTH_53) in the common ancestor of tetrapods, and the gain of the C-terminal CAAX-box (motif for prenylation and membrane attachment; C: cysteine, A: aliphatic amino acids, X: any amino acid) in the tetrapod lineages. On the viral side, our results reveal that the CpG bias among ssRNA viruses infecting different vertebrate groups ranges is variable, and it is not correlated to the host genome composition. RNA viruses infecting invertebrates, fish, and amphibians do not exhibit the strong CpG bias observed in viruses infecting mammals, reptiles, and birds. Among amniotes, this CpG bias is particularly pronounced in ssRNA viruses infecting mammals and squamate reptiles, while avian viruses display less consistent bias.

Overall, while mutation bias and genetic drift remain major evolutionary forces in viral genome evolution (Holmes 2009), our results suggest that host-specific selective pressures, particularly from the restriction factor ZAP, also play a significant role in shaping the dinucleotide composition of ssRNA viruses.

## Results

### ZAP Function Diverged From PARP12 More Than 400 Million Years Ago

To explore the evolutionary origins of ZAP-mediated recognition of CpG-rich RNA sequences, we searched for sequence homologs of PARP12 and ZAP in the genomes of representative species of the major vertebrate lineages (see supplementary Material and Methods, Supplementary material online). We seeded the searches with the PARP12 and ZAP sequences of humans and used the longest isoform as a representative transcript for each gene. We identified multiple gene candidates of homologs of PARP12 and/or ZAP in all vertebrate lineages, except for coelacanth and platypus. For the coelacanth, the single hit was identified as PARP12. In the case of platypus (*Ornithorhynchus anatinus*), searches seeded with the PARP12 (Q9H0J9) and ZAP (Q7Z2W4) amino acid sequences of humans retrieved the same protein: XP_028931066 (UPI0010A80474). Positions 1 to 691 of this protein match ZAP and the remainder matches PARP12. We considered this as an annotation artifact and manually split the sequence for all our downstream analyses.

**Fig. 1.**
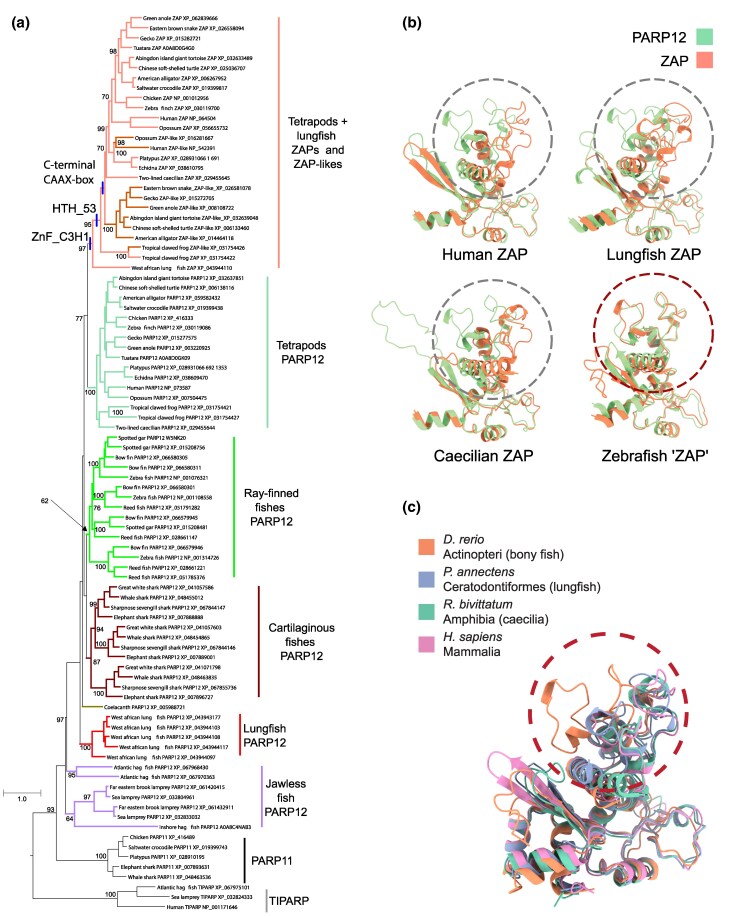
a) Maximum likelihood phylogram describing phylogenetic relationships among representative PARP12s, ZAPs, and ZAP-like genes of vertebrates. Vertical bands mark the inferred emergence of the HTH_53, ZnF_C3H1, and/or CAAX-box along the ZAP lineage. Ultrafast bootstrap Support values higher than 50% for nodes that define sets of orthologs, or identify duplications are shown. A version of the tree with support for every node is available as supplementary fig. S2, Supplementary Material online. b) Superimposition of the N-terminal domains of PARP12 (in green) and/or ZAP (in orange) from representative species of amniote, amphibian, lungfish, and bony fish. The positions shown for each structure superimpose with positions 1 to 228 from *H. sapiens* ZAP (Q7Z2W4). The dashed lines highlight the differences in the zinc-finger structure. Model confidence based on pLDDT scores for each structure is represented in supplementary fig. S3, Supplementary Material online. c) Superimposition of the N-terminal domains of ZAP for representative species of amniote, amphibian, lungfish, and bony fish. The dashed line highlights the differences in the zinc-finger structure between the bony fish and the other vertebrates.

We then estimated phylogenetic relationships among the sequences to reconstruct the duplicative history of the PARP12/ZAP gene family (Fig. 1a) using a small set of vertebrate PARP11 and TIPARP sequences as outgroup. Vertebrate members of the PARP12/ZAP gene family fall in a monophyletic group relative to TIPARP and PARP11. The deepest splits among vertebrates correspond to the jawless fish PARP12 sequences, which fall in 2 separate clades. Within jawed vertebrates, the deepest splits correspond to a group of lungfish PARP12 paralogs, followed by the single coelacanth PARP12 representative. The 3 separate PARP12 paralogs of cartilaginous fish fall sister to the PARP12s of ray-finned fishes, indicating that these 2 independent sets of duplications were lineage-specific. The ZAP genes of tetrapods fall sister to lungfish ZAP, and the ZAP clade is sister to the PARP12 genes of tetrapods.

The tree suggests that the PARP12 and ZAP genes of vertebrates follow contrasting evolutionary trajectories. Relationships among tetrapod PARP12 orthologs closely resemble the expected species tree except for a lineage-specific tandem duplication in the tropical clawed frog. By contrast, the ZAP portion of the tree reveals the presence of at least 3 additional ZAP-like clades, the first in the tropical clawed frog, the second one in sauropsids, and the third one in mammals (Fig. 1a). These 3 sets of genes lie in slightly different genomic contexts (supplementary fig. S1, Supplementary Material online), and forcing the ZAP-like genes to be monophyletic results in a significant loss in likelihood score (supplementary table S1, Supplementary Material online). These ZAP-like genes are significantly shorter than normal ZAPs, containing different domains: while human ZAP-like contains only the Zn-fingers, those of frogs and sauropsids possess the WWE and PARP domains (supplementary table S2, Supplementary Material online). Taken together, the evidence suggests these genes derive from independent duplications. Thus, this phylogeny indicates that the PARP12/ZAP gene family evolved following a birth-and-death model (Nei et al. 1997) because a reconciliation between the species tree and the gene tree uncovers multiple duplications and losses. In most cases, the different PARP12/ZAP paralogs lie within proximity on the same chromosome, suggesting that gains and losses are driven by ectopic recombination.

To further characterize PARP12/ZAP sequences, we predicted (i) the fold adopted by the zinc-finger domain, (ii) the presence of other conserved domains, and (iii) the presence of the CAAX-box in the C-terminus. We show that for ZAP of lungfish (*Dipnomorpha*) and amphibians (*Amphibia*), the zinc-finger superimposes with that of human ZAP, which has been reported to bind CpG-rich ssRNA, while bony fish orthologs do not structurally resemble the RBD of ZAP (Fig. 1b and c). This suggests that in a common ancestor to extant lungfishes and tetrapods, the zinc-finger acquired a new folding that likely resulted in a change in its binding specificity. We identified the presence of an additional HTH_53 domain in the ZAP sequences of tetrapods (amphibians, mammals, reptiles and birds), but not in lungfish (supplementary table S2, Supplementary Material online). Finally, we identified the presence of the CAAX-box in a few amphibian ZAP sequences and in all mammalian, reptile, and bird ZAP sequences, indicating that ZAP became able to anchor to cellular membranes in an early tetrapod (supplementary table S3, Supplementary Material online). The CAAX box in human ZAP may influence its antiviral activity through modification by cellular farnesyl transferases, which are conserved across eukaryotes (Stein et al. 2015).

Taken together, these results suggest that ZAP evolved from a PARP12-like ancestral protein through a process of neofunctionalization (Lynch and Conery 2000). Our analyses indicate that the structural and functional innovations that characterize ZAP were acquired in a stepwise manner over the course of its evolution (Fig. 1). This began in the last common ancestor of lungfish and tetrapods, ∼408 million years ago (Mya), with a PARP12-like duplicate that gradually acquired its antiviral properties. First, the duplicate gene evolved the ZAP type zinc-finger fold. Next, ZAP acquired the HTH_53 domain (supplementary table S2, Supplementary Material online), and finally, ZAP's ability to anchor to cellular membranes became widespread (supplementary table S3, Supplementary Material online).

### CpG Suppression in Amniote-Infecting Viruses Reflects Host Evolutionary Pressure

**Fig. 2.**
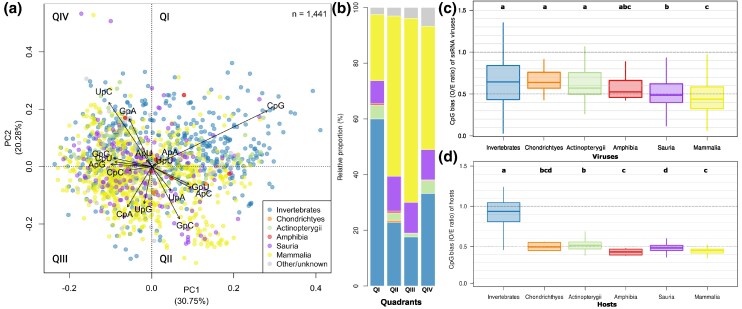
a) PCA of the dinucleotide bias of ssRNA viruses across metazoan groups. The PCA plot shows the dinucleotide bias (O/E ratio) of ssRNA viruses from different metazoan hosts. Each point represents an individual virus (*n* = 1,441), color coded according to its host group. The contributions of the first 2 principal components, PC1 and PC2, to the variability are 30.75% and 20.28%, respectively, accounting for a total of 51.03%. The vectors indicate the direction and relative magnitude of the original dinucleotide variables, indicating their contribution to the separation observed in the PCA plot. b) Quadrant analysis showing the relative proportion of ssRNA viruses per host in each PCA quadrant. Each PCA quadrant (Q) is defined as: Q1, PC1 > 0 and PC2 > 0; Q2, PC1 > 0 and PC2 < 0; Q3, PC1 < 0 and PC2 < 0; Q4, PC1 < 0 and PC2 > 0. The distribution of hosts varies significantly across the PCA quadrants (χ^2^, *P*-value < 2e^−16^). c) Comparative analysis of CpG bias in ssRNA viruses across different metazoan groups. The box plots illustrate the CpG bias (O/E ratio) in ssRNA viruses infecting different groups of vertebrates and invertebrates. The letters above the boxplots indicate statistically significant differences between groups according to multiple comparison tests (Wilcoxon rank sum test with Benjamini–Hochberg correction). Groups that do not share the same letter (a, b, c) are significantly different from each other (*P* ≤ 0.05). d) CpG bias distribution (O/E ratio) of different metazoans, with a more detailed subgrouping of the vertebrates. The letters above the boxplots indicate statistically significant differences between groups according to multiple comparison tests (Wilcoxon rank sum test with Benjamini–Hochberg correction). Groups that do not share the same letter (a, b, c, d) are significantly different from each other (*P* ≤ 0.05).

Given that ZAP has the ability to bind CpG-rich ssRNA viruses, we focused on the dinucleotide composition among these viruses. We performed a principal component analysis (PCA) on the dinucleotide bias (observed/expected (O/E) ratio) of all available reference viral genomes of ssRNA viruses (*n* = 1441) up to date that infect vertebrate or invertebrate metazoans (see Supplementary material and methods, Supplementary Material online and our Elsevier's Mendeley Data repository, available: https://doi.org/10.17632/knv6264vkr.1). The PCA illustrates the distribution of viral dinucleotide composition, colored according to their hosts (Fig. 2a), along the first 2 principal components (PC1 and PC2). The contributions of PC1 and PC2 to the variability are 30.75% and 20.28%, respectively, accounting for a total of 51.03%. The separation in dinucleotide composition between viruses infecting invertebrates and vertebrates highlights the heterogeneity in dinucleotide usage among metazoan ssRNA viruses. CpG emerges as a significant contributor to the PCA embedding, with both PC1 and PC2 capturing most variability associated with this dinucleotide (Fig. 2a and b). We then performed an analysis of CpG bias across ssRNA viruses infecting different animal hosts (Fig. 2c). The median CpG bias of viruses decreases with the phylogenetic proximity of their hosts to mammals. Viruses infecting invertebrates have the highest observed/expected CpG values, indicating minimal CpG suppression. Viruses infecting amphibians and fish (*Chondrichthyes* and *Actinopterygii*) show moderate CpG suppression. Amniote-infecting viruses (including those infecting mammals, birds, and reptiles) exhibit the strongest CpG suppression, with median CpG O/E values around 0.5. Due to the differing CpG bias among ssRNA viruses (Fig. 2c), we also investigated CpG bias in host genomes to uncover potential relationships (Fig. 2d). While invertebrate genomes appear overwhelmingly unbiased, all vertebrate genomes exhibit similar CpG bias, with median values around 0.5. This 2-fold difference between invertebrate and vertebrate genomes reflects the CpG methylation/deamination dynamics occurring in vertebrates. Therefore, host genomic mimicry might not be the only factor contributing to the compositional patterns observed in ssRNA viruses. These results are in agreement with the emergence of the catalytic antiviral function of ZAP.

## Discussion

Our study sheds light on the emergence and functional diversification of the ZAP family of genes. The ZAP genes emerged from the duplication of a PARP12-like gene over 400 million years ago in the common ancestor of lungfishes and tetrapods. Following this duplication, ZAP underwent significant structural changes, particularly in its zinc-finger domain, which exhibits binding affinity for CpG-rich ssRNA sequences, a key antiviral function (Fig. 1b). Our data suggest that the RBD of ZAP, which is structurally similar in lungfish, amphibians, and amniotes, can be traced back to their last common ancestor.

The ZAP gene family provides an excellent example of the neofunctionalization process on a macroevolutionary timescale. The evolution of ZAP can be broken into 3 steps: (i) duplication of the PARP12 gene in the common ancestor of lungfish and tetrapods, (ii) emergence of the characteristic ZAP zinc-finger fold in the common ancestor of lungfish and tetrapods, and (iii) acquisition of the CAAX box in a common ancestor of tetrapods, allowing endosomal/lysosomal targeting. The analysis of CpG suppression in ssRNA viruses (Fig. 2) highlights the influence of host genomic composition on viral evolution. To evade host immune detection and more efficiently hijack cellular components for replication, viruses are hypothesized to mimic the genomic composition of their host (Cheng et al. 2013). The pronounced CpG bias in vertebrate genomes, likely due to methylation and subsequent deamination, supports this hypothesis (Gentles and Karlin 2001; Raddatz et al. 2013). Importantly, PCA of dinucleotide bias reveals that viruses infecting amniotes exhibit the strongest CpG suppression, likely as an adaptation to avoid ZAP-mediated immune recognition.

Notably, the boxplots displaying CpG bias in ssRNA viruses (Fig. 2c) show greater variability compared to those of their hosts (Fig. 2d), suggesting that a range of biological and/or compositional factors may be contributing to this dispersion. Although the O/E ratio of dinucleotide frequencies helps normalize for base composition, particularly GC content, it does not fully account for higher-order sequence constraints or genome architecture. As a result, residual effects may remain despite controlling for GC content. Moreover, genome size, which varies widely among ssRNA viruses, can impact mutational biases and selective pressures, further affecting the CpG bias. Additionally, certain viral groups may evade ZAP activity, potentially by encoding antagonists that counteract its effects. Also, ZAP antiviral activity may be modulated in diverse ways by different hosts, tissues and cell-types (de Andrade and Cirne-Santos 2023).

The absence of KHNYN, a critical cofactor of ZAP, in birds (Lista et al. 2023) might be linked to the reduced CpG bias observed in avian viruses (supplementary fig. S4, Supplementary Material online). Avian and crocodilian viruses display lesser CpG bias than viruses infecting other reptiles and mammals. This observation suggests that avian ZAP, and by extension, crocodilian ZAP (where the hosts also lack KHNYN), exerts reduced selective pressure on the CpG content of infecting ssRNA viruses.

Unfortunately, the scarcity of viruses from other groups restricts a more detailed association between the presence/absence of ZAP. There is virtually no complete genome of viruses specific for coelacanths or lungfish.

Altogether, the evolution of ZAP involved both structural innovations and functional adaptations that match the predictions of the neofunctionalization model, and contributed to its antiviral role, while the CpG suppression observed in ssRNA viruses underscores the ongoing evolutionary arms race between hosts and their viral pathogens. These findings highlight the host-virus interactions and the evolutionary pressures that shape these dynamics.

## Supplementary Material

msaf135_Supplementary_Data

## Data Availability

All data used to produce our results are provided as supporting data and can be found in Elsevier's Mendeley Data repository, available at: https://doi.org/10.17632/knv6264vkr.1.
