# Supplementary material for: Impact of the Zinc Antiviral Protein on the Genomic Composition of RNA Viruses Infecting Vertebrates
Source: Mol Biol Evol. 2025 Jun 4;42(6):msaf135. doi: 10.1093/molbev/msaf135 (PMC12204182; doi:10.1093/molbev/msaf135)
Supplement: msaf135_Supplementary_Data [file msaf135_supplementary_data.zip › MBE-msaf135-Supplementary_Material.pdf]

SUPPLEMENTARY MATERIAL

Impact of the Zinc Antiviral Protein on the genomic composition of RNA viruses infecting vertebrates

Diego Simón[1,2,3#], Daniela Megrian[4#], Hunter K Walt[5], Pilar Moreno[1,2,6], Héctor Musto[3], Federico G Hoffmann[5,7] and Gonzalo Moratorio[1,2,6*]

1. Laboratorio de Virología Molecular, Facultad de Ciencias, Universidad de la República, Montevideo, Uruguay.
2. Laboratorio de Evolución Experimental de Virus, Institut Pasteur de Montevideo, Montevideo, Uruguay.
3. Laboratorio de Genómica Evolutiva, Facultad de Ciencias, Universidad de la República, Montevideo, Uruguay.
4. Unidad de Bioinformática, Institut Pasteur de Montevideo, Uruguay.
5. Department of Biochemistry, Nutrition and Health Promotion, Mississippi State University, Mississippi State, MS 39762, USA.
6. Centro de Innovación en Vigilancia Epidemiológica, Institut Pasteur de Montevideo, Montevideo, Uruguay.
7. Institute for Genomics, Biocomputing and Biotechnology, Mississippi State University, Mississippi State, MS 39762, USA.

[#] These authors contributed equally to this work.

* Corresponding Author: E-mail: moratorio@pasteur.edu.uy

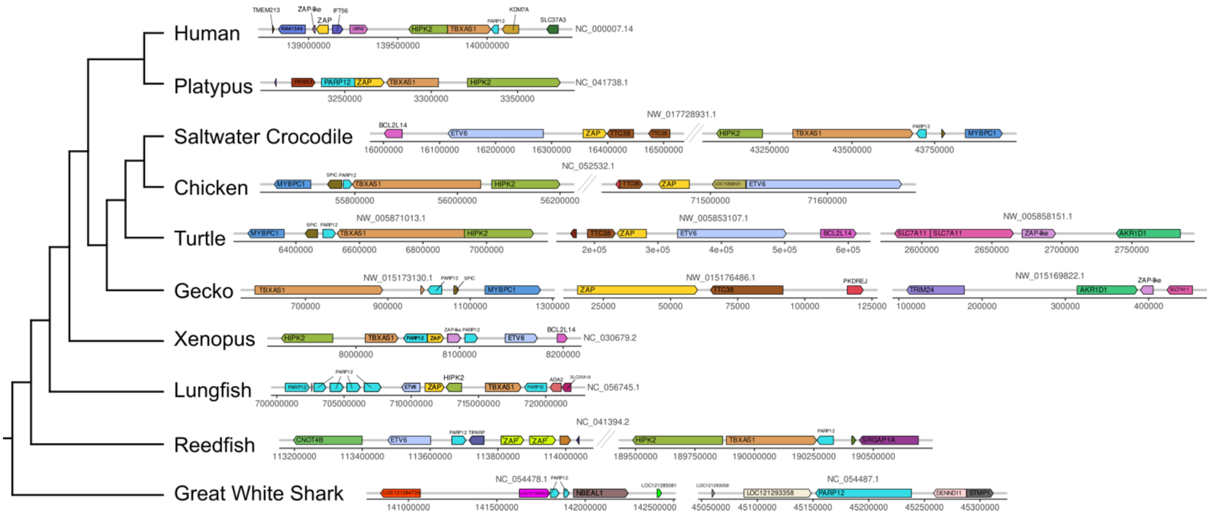

Supplementary Figure 1. Genomic context of PARP12, ZAP, and ZAP-like genes across the vertebrate phylogeny. Orthologous genes have the same labels and color.

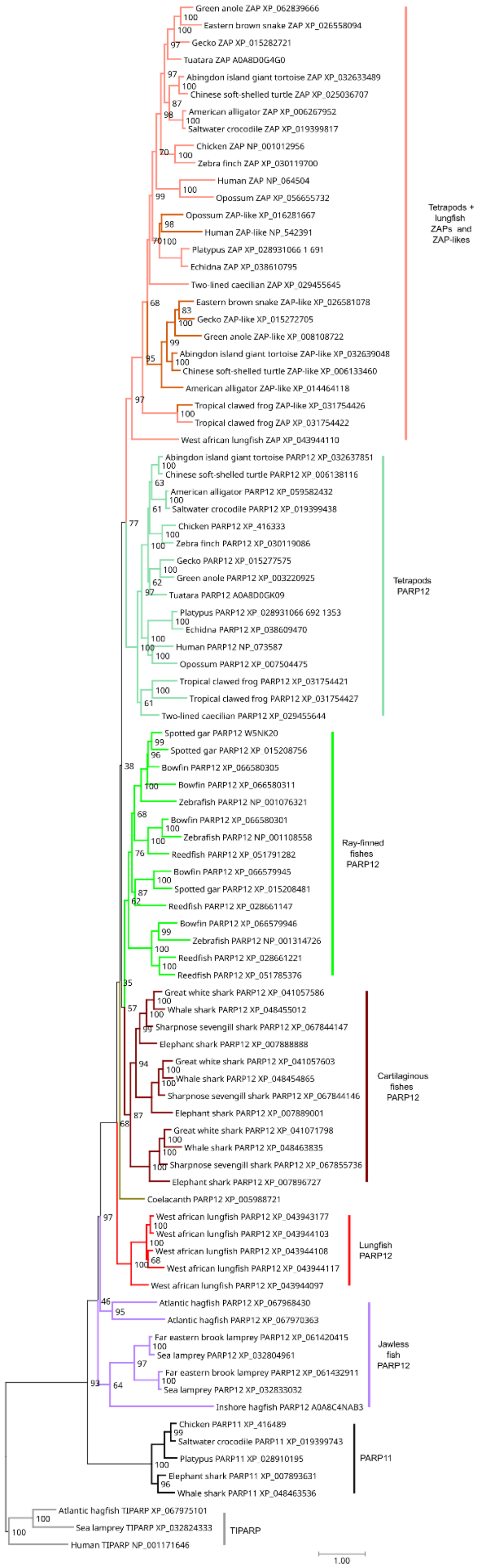

Supplementary Figure 2. Maximum likelihood phylogram describing phylogenetic relationships among representative PARP12s, ZAPs, and ZAP-like genes of vertebrates. Ultrafast bootstrap Support values for every node are shown.

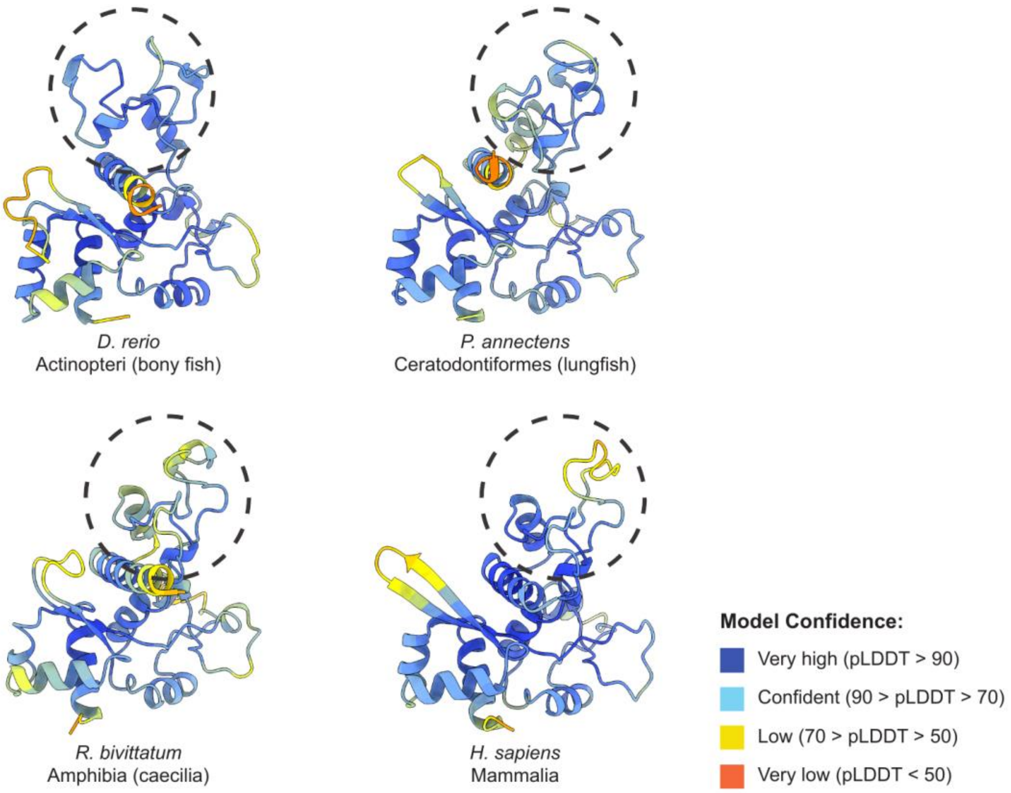

Supplementary Figure 3. Predicted 3D structures of the N-terminal domain of ZAP orthologs in representative vertebrates, generated with AlphaFold. The positions shown for each structure superimpose with positions 1-228 from H. sapiens ZAP (Q7Z2W4). Colour scale indicates model confidence based on pLDDT scores.

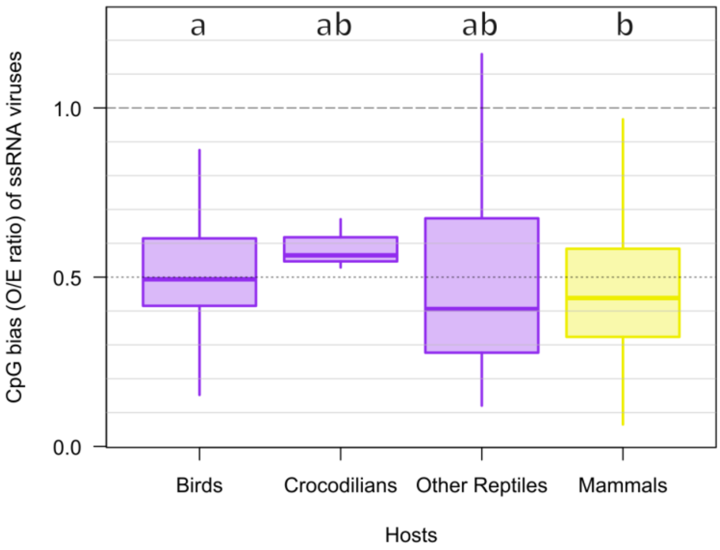

Supplementary Figure 4. CpG bias (observed/expected) for ssRNA viruses infecting squamate reptiles, crocodilians, birds and mammals. The letters above the boxplots indicate statistically significant differences between groups according to multiple comparison tests (Wilcoxon rank sum test with Benjamini-Hochberg correction). Groups that do not share the same letter (a, b, c) are significantly different from each other ($p \leq 0.05$).

Materials and Methods

Vertebrates database assembly

To carry out a comprehensive analysis of ZAP, we surveyed 30 genomes representing the major lineages of vertebrates available in NCBI Genome databases (Sayers et al. 2024) or Ensembl (Harrison et al. 2024), last accessed in October 2024. Because ZAP is only known from amniotes our species choice focused on this group. We surveyed the genomes of 4 cyclostomes (*Cyclostomata*), 4 cartilaginous fishes (*Chondrichthyes*), 4 ray-finned fishes (*Actinopterygii*), 1 coelacanth (*Coelacanthiformes*), 1 lungfish (*Dipnomorpha*), 2 amphibians (*Amphibia*), 8 reptiles (1 rhynchocephalian [*Sphenodontia*], 3 squamates [*Squamata*], 2 *Testudines*, 2 crocodilians [*Crocodylia*]), 2 birds (*Aves*), and 4 mammals (*Mammalia*).

To identify PARP12/ZAP homologs, orthologs of human PARP12 (Q9H0J9) and ZAP (Q7Z2W4) from all previously mentioned vertebrate lineages were downloaded from NCBI or Ensembl database. The canonical transcripts were preferred, identified using gget info, and their amino acid sequences were obtained using gget seq in translate mode (Luebbert and Pachter 2023). The PARP12 ortholog from each species was then used to BLAST against its own proteome to ensure all PARP12, ZAP, and ZAP-like (Q96H79) paralogs were collected. If a vertebrate taxon was not available in Ensembl, we identified PARP12 and ZAP orthologs by querying the human PARP12 protein against the proteome of the taxon of interest using BLASTp. All PARP12, ZAP, and ZAP-like proteins were collected, and their gene names were identified using NCBI datasets to download their gene data package (O'Leary et al. 2024). Each gene was represented by its longest protein isoform for phylogenetic analyses.

Genomic context analyses

To examine the genomic context of PARP12, ZAP, and ZAP-like genes across the vertebrate phylogeny, we retrieved the genomic coordinates, orientation, and gene names of 2 protein-coding genes flanking upstream and downstream of each PARP12, ZAP, and ZAP-like gene using their gff annotation files and a custom bash script. These analyses were restricted to jawed vertebrates and included a single species per lineage with the exception of mammals, where we included platypus and human. Each gene was represented by its NCBI gene name, however, genes with ambiguous names (i.e. LOC identifiers) were renamed if they were homologous to other flanking genes in our analysis based on reciprocal best BLAST hits using

their protein sequences. The location of each gene along its chromosome was drawn using the gggenes v0.5.1 package (Wilkins 2023) implemented in R.

Phylogenetic analyses

We aligned the PARP12 and ZAP amino acid sequences with 3 TIPARP and 5 PARP11 outgroup sequences using the L-ins-I algorithm of MAFFT v7.505 (Katoh and Standley 2013). The resulting alignment was then used to estimate phylogenetic relationships using maximum likelihood as implemented in IQTREE2 v.2.0.7 (Nguyen et al. 2015; Minh et al. 2020). We used the ModelFinder routine (Kalyaanamoorthy et al. 2017) to select the best-fitting amino acid substitution model identified by the Bayesian Information Criterion (JTT+R6). Branch support was assessed using 10000 pseudoreplicates of ultrafast bootstrap (Hoang et al. 2018). Alternative phylogenetic hypotheses were compared using the approximately unbiased test (Shimodaira 2002) as implemented in IQ-Tree. The alignments, tree files, and a log of the commands required to replicate our results can be found in Elsevier's Mendeley Data repository, available: doi.org/10.17632/knv6264vkr.1. Divergence times were retrieved from TimeTree (Kumar et al. 2022).

Domain based annotations

Functional domain annotation of protein sequences was performed using InterProScan v5.71-102.0 (Jones et al. 2014), restricted to Pfam database (Mistry et al. 2021).

Protein structure prediction and analysis

We predicted the protein structure of a PARP12 and a ZAP representative sequence for each taxonomic class using AlphaFold 2 (Jumper et al. 2021). We compared PARP12 and ZAP domain structures using the matchmaker command of ChimeraX (Pettersen et al. 2021). All protein structure figures were generated with ChimeraX (Pettersen et al. 2021).

Viral dataset construction

Viral diversity was accessed following the International Committee on Taxonomy of Viruses (ICTV) resources (Lefkowitz et al. 2018). ICTV provides a downloadable spreadsheet of all recognized viral species on its Master Species List (MSL; available at ictv.global/msl). From the current version (MSL39), all single-stranded RNA (ssRNA) viruses were considered, including:

positive-sense, ssRNA(+); negative-sense, ssRNA(-); ambisense ssRNA(+/-); and retro-transcribing (RT), ssRNA-RT.

After categorizing the ssRNA viruses, we selected only those with NCBI Reference Sequence (RefSeq) genome(s) available. Viral genome assemblies were accessed from RefSeq Release 222 (available as of January 16, 2024, from ftp.ncbi.nlm.nih.gov/genomes/refseq/viral/).

Virus and host relationships as available at Virus-Host DB, which organizes data as pairs of viruses and their hosts (Mihara et al. 2016). A total 1441 ssRNA viral genomes were, including only viruses infecting metazoans. This study covers a wide spectrum of viruses from vertebrates (n = 975; i.e., 4 *Chondrichthyes*, 47 *Actinopterygii*, 6 *Amphibia*, 153 *Sauria*, 707 *Mammalia*, and 60 other or unknown vertebrate hosts) and invertebrates (n = 466). To increase taxonomic diversity beyond the RefSeq dataset, additional complete genomes were retrieved from GenBank (Clark et al. 2016) for underrepresented groups: 1 *Chondrichthyes* (BK063518) and 3 *Amphibia*. We also included 3 complete viral genomes annotated as crocodilian ssRNA viruses (*Crocodylia*: LC817237, PQ373863, PQ869318; out of 4 in total, one removed [PQ869319] for redundancy, as being the same viral species as PQ869318). These genomes were reanalysed using the same approach previously described.

Dinucleotide composition

The relative numbers of each nucleotide and dinucleotide (i.e., each pair of adjacent nucleotides) were computed (see data availability below). The dinucleotide frequencies computed (from here on, observed frequencies) are often compared to the expected frequencies to detect deviations that could reveal underlying biological mechanisms or constraints. The dinucleotide bias was calculated as the observed frequency of a given dinucleotide (XY), over the expected frequency based on the frequency of each nucleotide that constitutes that dinucleotide (X and Y). In display style formulas, it could be written as follows:

$$\text{Dinucleotide bias (O/E ratio)} = \frac{XY}{X \cdot Y}$$

It is to be remarked that the expected frequency of the dinucleotide XY is exactly the same as the expected frequency of the dinucleotide YX, but this equivalence is not necessarily true for the observed frequencies. Furthermore, an observed/expected (O/E) ratio close to 1 means that there is no apparent bias, whereas a value away from 1 implies a bias; values well below

1 suggest underrepresentation of the dinucleotide, and values well above 1 suggest overrepresentation.

Host genomic composition was assessed from DNA HIVE database (Alexaki et al. 2019; available at dnahive.fda.gov/dna.cgi?cmd=codon_usage&id=537&mode=cocoputs), for a total of 653 hosts (242 invertebrates and 411 vertebrates [5 *Chondrichthyes*, 119 *Actinopterygii*, 8 *Amphibia*, 163 *Sauria*, and 116 *Mammalia*]).

Principal component analysis

Principal component analysis (PCA) was performed using the R package vegan, version 2.6-4 (Oksanen et al. 2022) a robust statistical method widely employed for dimensionality reduction and visualization of high-dimensional datasets. PCA allows for the identification of key patterns and relationships within the data by transforming variables into orthogonal components, thereby facilitating the exploration of underlying structures and trends.

Box plots and statistical significance

To visualize the distribution of dinucleotide bias of viruses and hosts we employed box plots, generated using the boxplot function available in the base R package (R Core Team 2024). It is important to note that the groups are not evenly distributed in terms of sample size (e.g., ranging from few viral and host genomes from *Chondrichthyes* to hundreds from *Sauria* or *Mammalia*). Such differences in the availability of data for certain taxa are due to biases in the databases from which our data were sourced.

To test statistically significant differences between multiple groups we use the Kruskal-Wallis rank sum test. This was followed up with post hoc pairwise comparisons using the Wilcoxon rank sum test with continuity correction. For multiple testing comparisons, the *p*-values have been adjusted using the Benjamini-Hochberg correction.

References

Alexaki A, Kames J, Holcomb DD, Athey J, Santana-Quintero LV, Lam PVN, Hamasaki-Katagiri N, Osipova E, Simonyan V, Bar H, Komar AA, Kimchi-Sarfaty C. 2019. Codon and Codon-Pair Usage Tables (CoCoPUTs): Facilitating Genetic Variation Analyses and Recombinant Gene Design. *J Mol Biol.* 431(13):2434–2441. https://doi.org/10.1016/j.jmb.2019.04.021

Clark K, Karsch-Mizrachi I, Lipman DJ, Ostell J, Sayers EW. 2016. GenBank. *Nucleic Acids Res*. 44:D67–D72. https://doi.org/10.1093/nar/gkv1276

Harrison PW, Amode MR, Austine-Orimoloye O, Azov AG, Barba M, Barnes I, Becker A, Bennett R, Berry A, Bhai J, et al. 2024. Ensembl 2024. *Nucleic Acids Res*. 52(D1):D891–D899. https://doi.org/10.1093/nar/gkad1049

Hoang DT, Chernomor O, von Haeseler A, Minh BQ, Vinh LS. 2018. UFBoot2: Improving the ultrafast bootstrap approximation. *Mol Biol Evol.* 35(2):518–522. https://doi.org/10.1093/molbev/msx281

Jones P, Binns D, Chang HY, Fraser M, Li W, McAnulla C, McWilliam H, Maslen J, Mitchell A, Nuka G, et al. 2014. InterProScan 5: genome-scale protein function classification. *Bioinformatics*. 30(9):1236–1240. https://doi.org/10.1093/bioinformatics/btu031

Jumper J, Evans R, Pritzel A, Green T, Figurnov M, Ronneberger O, Tunyasuvunakool K, Bates R, Žídek A, Potapenko A, et al. 2021. Highly accurate protein structure prediction with AlphaFold. *Nature*. 596(7873):583–589. https://doi.org/10.1038/s41586-021-03819-2

Kalyaanamoorthy S, Minh BQ, Wong TKF, von Haeseler A, Jermiin LS. 2017. ModelFinder: fast model selection for accurate phylogenetic estimates. *Nat Methods*. 14(6):587–589. https://doi.org/10.1038/nmeth.4285

Katoh K, Standley DM. 2013. MAFFT multiple sequence alignment software version 7: improvements in performance and usability. *Mol Biol Evol*. 30(4):772–780. https://doi.org/10.1093/molbev/mst010

Kumar S, Stecher G, Suleski M, Hedges SB. 2017. TimeTree: A resource for timelines, timetrees, and divergence times. *Mol Biol Evol*. 34(7):1812–1819. https://doi.org/10.1093/molbev/msx116

Lefkowitz EJ, Dempsey DM, Hendrickson RC, Orton RJ, Siddell SG, Smith DB. 2018. Virus taxonomy: the database of the International Committee on Taxonomy of Viruses (ICTV). *Nucleic Acids Res*. 46(D1):D708–D717. https://doi.org/10.1093/nar/gkx932

Luebbert L, Pachter L. 2023. Efficient querying of genomic reference databases with gget. *Bioinformatics*. 39(1):btac836. https://doi.org/10.1093/bioinformatics/btac836

Mihara T, Nishimura Y, Shimizu Y, Nishiyama H, Yoshikawa G, Uehara H, Hingamp P, Goto S, Ogata H. 2016. Linking virus genomes with host taxonomy. *Viruses*. 8(3):66. https://doi.org/10.3390/v8030066

Minh BQ, Schmidt HA, Chernomor O, Schrempf D, Woodhams MD, von Haeseler A, Lanfear R. 2020. IQ-TREE 2: New models and efficient methods for phylogenetic inference in the genomic era. *Mol Biol Evol*. 37(5):1530–1534. https://doi.org/10.1093/molbev/msaa015

Mistry J, Chuguransky S, Williams L, Qureshi M, Salazar GA, Sonnhammer ELL, Tosatto SCE, Paladin L, Raj S, Richardson LJ, et al. 2021. Pfam: The protein families database in 2021. *Nucleic Acids Res*. 49:D412–D419. https://doi.org/10.1093/nar/gkaa913

Nguyen LT, Schmidt HA, von Haeseler A, Minh BQ. 2015. IQ-TREE: a fast and effective stochastic algorithm for estimating maximum-likelihood phylogenies. *Mol Biol Evol*. 32(1):268–274. https://doi.org/10.1093/molbev/msu300

Oksanen J. 2022. vegan: Community Ecology Package. CRAN. https://cran.r-project.org/package=vegan

O'Leary NA, Cox E, Holmes JB, Anderson WR, Falk R, Hem V, Tsuchiya MTN, Schuler GD, Zhang X, Torcivia J, et al. 2024. Exploring and retrieving sequence and metadata for species across the tree of life with NCBI Datasets. *Scientific Data.* 11(1):732. https://doi.org/10.1038/s41597-024-03571-y

Pettersen EF, Goddard TD, Huang CC, Meng EC, Couch GS, Croll TI, Morris JH, Ferrin TE. 2021. UCSF ChimeraX: Structure visualization for researchers, educators, and developers. *Protein Sci*. 30(1):70–82. https://doi.org/10.1002/pro.3943

R Core Team. 2024. R: A Language and Environment for Statistical Computing. R Foundation for Statistical Computing, Vienna, Austria. https://www.r-project.org/

Sayers EW, Beck J, Bolton EE, Brister JR, Chan J, Comeau DC, Connor R, DiCuccio M, Farrell CM, Feldgarden M, et al. 2024. Database resources of the National Center for Biotechnology Information. *Nucleic Acids Res*. 52(D1):D33–D43. https://doi.org/10.1093/nar/gkad1044

Shimodaira H. 2002. An approximately unbiased test of phylogenetic tree selection. *Syst Biol*. 51(3):492–508. https://doi.org/10.1080/10635150290069913

Wilkins D. 2023. gggenes: DrWilkins D. 2023. gggenes: Draw Gene Arrow Maps in 'ggplot2'. R package version 0.5.0. https://wilkox.org/gggenes/aw Gene Arrow Maps in 'ggplot2'. R package version 0.5.0. https://wilkox.org/gggenes/
