# Supplementary material for: Impact of the Zinc Antiviral Protein on the Genomic Composition of RNA Viruses Infecting Vertebrates
Source: Mol Biol Evol. 2025 Jun 4;42(6):msaf135. doi: 10.1093/molbev/msaf135 (PMC12204182; doi:10.1093/molbev/msaf135)
Supplement: msaf135_Supplementary_Data [file msaf135_supplementary_data.zip › MBE-msaf135-Supplementary_Table_1.pdf]

Supplementary Table 1. Statistical comparisons of constrained trees

Tree	logL	deltaL	bp-RELL	p-KH	p-SH	c-ELW	p-AU
1	-83259.13	202.1	0	0	0	1.01e-41	5.24e-55
2	-83057.07	0	1	1	1	1	1

Tree 1: tetrapod ZAP-likes monophyletic and sister to tetrapod ZAPs
Tree 2: unconstrained maximum likelihood tree (figure 1)

deltaL: logL difference from the maximal logl in the set.
bp-RELL: bootstrap proportion using RELL method (Kishino et al. 1990).
p-KH: p-value of one sided Kishino-Hasegawa test (1989).
p-SH: p-value of Shimodaira-Hasegawa test (2000).
c-ELW: Expected Likelihood Weight (Strimmer & Rambaut 2002).
p-AU: p-value of approximately unbiased (AU) test (Shimodaira, 2002).
